# Preliminary evidence for performance enhancement following parietal lobe stimulation in Developmental Dyscalculia

**DOI:** 10.3389/fnhum.2014.00038

**Published:** 2014-02-07

**Authors:** Teresa Iuculano, Roi Cohen Kadosh

**Affiliations:** ^1^Department of Experimental Psychology, University of OxfordOxford, UK; ^2^Stanford Cognitive and Systems Neuroscience Laboratory, Department of Psychiatry and Behavioral Sciences, Stanford University School of MedicinePalo Alto, CA, USA; ^3^Institute of Cognitive Neuroscience, University College LondonLondon, UK

**Keywords:** transcranial electrical stimulation, learning, rehabilitation, neural compensation, Developmental Dyscalculia

## Abstract

Nearly 7% of the population exhibit difficulties in dealing with numbers and performing arithmetic, a condition named Developmental Dyscalculia (DD), which significantly affects the educational and professional outcomes of these individuals, as it often persists into adulthood. Research has mainly focused on behavioral rehabilitation, while little is known about performance changes and neuroplasticity induced by the concurrent application of brain-behavioral approaches. It has been shown that numerical proficiency can be enhanced by applying a small—yet constant—current through the brain, a non-invasive technique named transcranial electrical stimulation (tES). Here we combined a numerical learning paradigm with transcranial direct current stimulation (tDCS) in two adults with DD to assess the potential benefits of this methodology to remediate their numerical difficulties. Subjects learned to associate artificial symbols to numerical quantities within the context of a trial and error paradigm, while tDCS was applied to the posterior parietal cortex (PPC). The first subject (DD1) received anodal stimulation to the right PPC and cathodal stimulation to the left PPC, which has been associated with numerical performance's improvements in healthy subjects. The second subject (DD2) received anodal stimulation to the left PPC and cathodal stimulation to the right PPC, which has been shown to impair numerical performance in healthy subjects. We examined two indices of numerical proficiency: (i) automaticity of number processing; and (ii) mapping of numbers onto space. Our results are opposite to previous findings with non-dyscalculic subjects. Only anodal stimulation to the left PPC improved both indices of numerical proficiency. These initial results represent an important step to inform the rehabilitation of developmental learning disabilities, and have relevant applications for basic and applied research in cognitive neuroscience, rehabilitation, and education.

## Introduction

Poor numerical skills are a more severe handicap than most people realize. A recent cohort study on the effects of low numeracy shows that “it is more of a handicap in the workplace than poor literacy” (Bynner and Parsons, 1997), and individuals with poor numerical skills “are more than two and half times as likely to be unemployed, and more than three and half time as likely to be depressed” (Parsons and Bynner, 2005). Notably, it has been reported that if compared to their numerically competent peers, about half of them are unemployed by the age of 30 and twice as many are in poor physical health (Parsons and Bynner, 2005).

A major cause of poor numerical skills is Developmental Dyscalculia (DD) which, according to the current best estimates, affects about 3–7% of the population (Butterworth and Reigosa-Crespo, 2007; Shalev, 2007; Reigosa-Crespo et al., 2012). The usual presenting symptoms of DD are poor performance in school math tests, failing to understand numerical concepts, losing track in math lessons, often in the presence of good marks in other school subjects, and even more crucially, inability to deal with numbers in everyday life situations such as paying bills, telling the time, and remembering phone numbers. Notably, it has been noted that DD could persist into adulthood. In a 6-year prospective follow-up study, Shalev et al. showed that of the learners diagnosed as DD at age 11, over 40% were still in the DD category—i.e., 2 years behind the control population according to their criteria—at age 17, and 95% were still in the lowest quartile of their age group (Shalev et al., 2005). These reports highlight the crucial need to develop efficient ways to improve math performance in individuals with DD.

The underlying causes of DD are largely unknown yet, neural aberrancies in the volumetric and functional aspects of the posterior parietal cortex (PPC) have long been posited as the potential neurobiological origin of the disorder (Butterworth et al., 2011). A seminal study reports that adolescents of very low birth weight who showed deficits in math as determined by standardized tests had reduced gray matter volume in the left PPC, specifically in the intra-parietal sulcus (IPS) (Isaacs et al., 2001). However, both left and right volumetric aberrancies in the PPC have been reported—see for example (Rotzer et al., 2008).

Concurrent with the structural abnormalities, functional aberrancies have been reported in the posterior aspect of the parietal cortex of individuals with DD (Kucian et al., 2011), reflecting a differential modulation of this area in response to numerical stimuli in these individuals (Price et al., 2007). Critically, dyscalculic*-like* performance can be elicited in healthy adults following the application of a non-invasive brain stimulation technique that interrupts normal neuronal functions (i.e., transcranial magnetic stimulation -TMS) to the right PPC (Cohen Kadosh et al., 2007a). Moreover, the right PPC seems to be responsible for the intact development of numerical skills already during infancy (Hyde et al., 2010), and early childhood (Ansari et al., 2005; Cantlon et al., 2006). Importantly, recent studies have reported activity modulation of the right and left PPC as a function of stimulus's difficulty over development (Ansari et al., 2005; Rivera et al., 2005; Kaufmann et al., 2011). Moreover, it has been suggested that later in development the left PPC starts being recruited for the successful mastering of more refined numerical computations that are the product of enculturation, such as magnitude comparisons with numerical symbols (Ansari et al., 2005; Ansari and Dhital, 2006; Ansari, 2008). Altogether, these studies seem to suggest that aberrancies in parietal lobe systems are related to immature and/or poor numerical proficiency.

In parallel, cognitive and developmental studies indicate that the automatic processing of quantity—as reflected by Stroop-like effects in a numerical Stroop paradigm—is related to better and more mature numerical proficiency (Tzelgov et al., 1992; Girelli et al., 2000; Rubinsten et al., 2002; Schwarz and Ischebeck, 2003). Specifically, in these types of paradigms subjects are presented with two stimuli expressed as numerical digits and are required to compare them according to their physical size. A common finding is that incongruent trials are slower to be processed than congruent trials (congruity effect). This effect has been interpreted as an indicator that the subject processes numbers automatically even when the task does not require so, and in this sense has been considered a reliable index of numerical proficiency. This idea has been corroborated by the findings that adults with DD (Rubinsten and Henik, 2006) and typically developing children in their first year of school (Girelli et al., 2000; Rubinsten et al., 2002) do not seem to show this effect. Another signature of numerical proficiency has been identified in the accurate mapping of numbers to space. Notably, proficient numerical abilities are characterized by a linear mapping of numbers to space (Booth and Siegler, 2008; Dehaene et al., 2008).

In the present study we used transcranial direct current stimulation (tDCS), the most common application of transcranial electrical stimulation (tES) to affect numerical competence in two adult individuals with DD. tDCS applies low-amplitude direct currents via scalp electrodes, which penetrate the skull to enter the brain, thus modifying the trans-membrane neuronal potential and thereby influencing the level of excitability and modulating the firing rate of individual neurons in response to given inputs (Wagner et al., 2007; Paulus, 2011; Marquez-Ruiz et al., 2012; Cohen Kadosh, 2013). tDCS affects behavioral performance depending on the type of stimulation—i.e., anodal stimulation enhances performance, while cathodal stimulation impairs it.

Importantly, tDCS was coupled with a numerical learning paradigm (Cohen Kadosh et al., 2010) which used artificial digits—i.e., the Gibson figures (Gibson et al., 1962)—to investigate the development of numerical automaticity and the interaction between numbers and space in these two DD individuals. Numerical automaticity was assessed by measuring the congruity effect in a Stroop-*like* task (see Tzelgov et al., 1992; Girelli et al., 2000; Rubinsten et al., 2002; Schwarz and Ischebeck, 2003), while the interaction between numbers and space was tested via a task that required the subject to estimate the location of a given value on a number line (Iuculano and Butterworth, 2011). As mentioned above, numerical automaticity and the accurate mapping of numbers onto space are two well-documented behavioral signatures of numerical proficiency (Girelli et al., 2000; Rubinsten et al., 2002; Rubinsten and Henik, 2006; Booth and Siegler, 2008; Dehaene et al., 2008). Thus, the aim of the present study was to test whether the application of tDCS to the PPC, a key neural hub for the efficient processing of numerical information, can affect these basic numerical abilities in our two DD individuals.

## Materials and methods

### Participants

Two right-handed English speaking DD adults took part in the study (both females; mean age: 29.5 years, *SD* = 4.95). They were diagnosed with DD on the basis of the *Dyscalculia Screener* (Butterworth, 2003), an additional standardized arithmetical task—the Graded Difficulty Arithmetical (GDA) test—(Jackson and Warrington, 1986), and the Arithmetical subtest of the Wechsler Adult Intelligence Scale (WAIS) (Wechsler, 1986). DD participants also undertook additional domain-general assessments to test general intelligence (Wechsler, 1986), and were also tested on a non-symbolic number comparison task, a key test of intact number processing (Halberda et al., 2008, 2012). To be classified as dyscalculic, participants had to obtain: (i) a standardized score below 81 on at least one of the two tasks of the “Capacity subscale” of the *Dyscalculia Screener* (*see below*), for which the test average of the nationally standardized score = 100, *SD* = 15; (ii) an IQ score within the normal range (full-scale IQ not below 80); and (iii) impaired performance on the GDA test and the Arithmetical subtest of the WAIS. Both participants met our inclusion criteria (see Table 1). Please also note that on the non-symbolic number comparison task, both participants exhibited a very rudimentary performance, as indicated by a high Weber Fraction (WF) (Piazza et al., 2010). Importantly none of the participants had deficits in their visuo-spatial reasoning as measured by the Block Design subtest of the WAIS (see Table 1). Finally, none of the participants reported significant neurological or psychiatric disorders.

**Table 1:** **Demographic, IQ, diagnostic and experimental measures of the two DD individuals**.

**Measure**	**Individual DD**	
	***DD1***	***DD2***
Age (years)	33	26
**DOMAIN-GENERAL ASSESSMENTS**		
**IQ – WAIS scale**		
Full IQ	92	118
Verbal IQ	91	114
Performance IQ	96	121
***Visuo-spatial skills-WAIS***		
Block design^a^	13	15
**DOMAIN-SPECIFIC ASSESSMENTS**		
**Dyscalculia screene^b^**		
Simple RTs	**1**	4
***Capacity subscale***	3.5	**2.5**
Dot enumeration	**1**	4
Number comparison	8	**1**
***Achievement subscale***	**1**	3.5
Addition	**1**	4
Multiplication	**1**	**3**
GDA^c^	8 [**3**]	9 [**3**]
**Non-symbolic number comparison**		
WF^d^	**0.64**	**0.26**
***Arithmetical test - WAIS***		
Arithmetic	**50%ile**	**50%ile**

a Individual scores. Median centred at 10, with a standard deviation of 2.5.

b Stanine scores ranging from 1 to 9 whereby the better the performance the higher the stanine score (see Butterworth, 2003) [scores 0 to 3: low average; scores 4 to 6: average; scores 7 to 9: high average].

c Graded Difficulty Arithmetic Test (Jackson and Warrington, 1986). Scaled-score and correspondent level of performance in brackets [3 = dull average].

d WF, Weber Fraction, which constitutes another sensitive index of numerical proficiency (see Halberda et al., 2008, 2012; Piazza et al., 2010). Please note that the WF values reported by our two individuals with DD are very high. Neurotypical adults normally present a WF between 0.11 (Halberda et al., 2008) and 0.15 (Piazza et al., 2010). Notably, the values reported by these DD individuals are instead similar to the performance expected from 10 year olds, and pre-schoolers (DD2) and/or children with Developmental Dyscalculia (DD1) (Piazza et al., 2010). Impaired performance is shown in bold.

The study was approved by the local ethics committee and informed written consent was obtained for every subject before the start of each session.

### The dyscalculia screener

The *Dyscalculia Screener* (Butterworth, 2003) is a standardized computer-based test that comprises a total of four item-timed tasks. These four tasks are divided into two subscales: (i) *Capacity subscale*, which involves a dot enumeration task and a number comparison task, and (ii) *Achievement subscale*, which involves two arithmetic tasks, namely addition and multiplication both characterized by a verification-type format (Butterworth, 2003; Iuculano et al., 2008).

### Experimental investigation

#### Learning task

**Figure 1:**
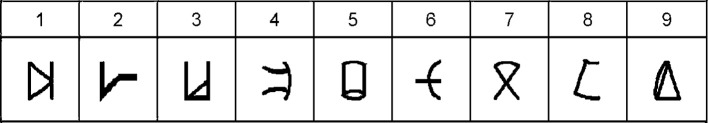
**Artificial digits**. Symbols used as stimuli during the learning phase and the numerical Stroop task and their equivalent as everyday digits*—*adapted from Tzelgov et al. (2000). Reprinted from Cohen Kadosh et al. (2010), with permission from Elsevier.

For this task we used nine artificial digits—Gibson figures (Gibson et al., 1962)—which were arbitrarily assigned to the numbers 1–9 (Figure 1) and used as stimuli.

**Figure 2:**
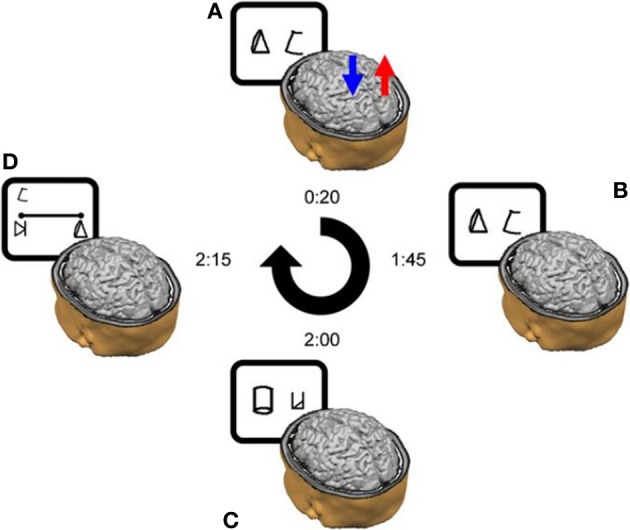
**Schematic outline of the experimental design in a typical daily session. (A)** tDCS was delivered for 20 min from the start of the training. In this example, anodal stimulation is applied to the right parietal lobe (red arrow), whereas cathodal stimulation is delivered to the left parietal lobe (blue arrow). **(B)** The training continued after the termination of the stimulation. **(C)** Once the training ended, the subjects performed the numerical Stroop task and **(D)** the number line task. The time next to each image reflects the elapsed time from the beginning of the daily session until its termination in a cumulative fashion. Please note that on Day 1 only, the session ended after the learning phase—thereby it did not include the experimental tasks (i.e., Stroop-task and number line task). Reprinted from Cohen Kadosh et al. (2010), with permission from Elsevier.

Subjects were instructed to refer to the meaningless symbols (i.e., the artificial digits) as representing various magnitudes. Each trial began with a fixation point (in white ink) for 300 ms at the center of a black computer screen. 300 ms after the fixation disappeared two symbols (vertical visual angle of 2.63°) appeared on the computer screen, one symbol in the left visual field, and another in the right visual field. The center-to-center distance between the two digits subtended a horizontal visual angle of 9.7°. The symbol pair appeared and remained in view until the participant pressed a key (but not for more than 5 s). Visual feedback (“Correct Answer”/“Mistake”/“No Response”) was provided for every trial for 500 ms. A new trial began 200 ms after the feedback. Each learning session was divided into 11 blocks of trials, each block consisting of 144 symbol pair comparisons (trials) that included 18 comparisons for each adjacent pair (e.g., 1–2, 2–3, 3–4, etc.). The presentation in each block appeared in a random order. A training block with 48 trials was performed at the beginning of the task. Participants were instructed to choose the symbol they thought had a larger magnitude in each pair. They were asked to respond as quickly as possible but to avoid mistakes, and to indicate their choices by pressing one of two keys (i.e., P or Q on the keyboard) corresponding to the side of the display with the selected member of the digit pair. The right answer appeared equal times on the right and left sides and all pairs appeared equally often. Participants were provided with the average reaction time of the correct answers and percentage of errors after one third, two thirds and the end of each block. The learning task was the first task to be done in all six sessions (Figures 2A,B).

#### Experimental tasks

**Figure 3:**
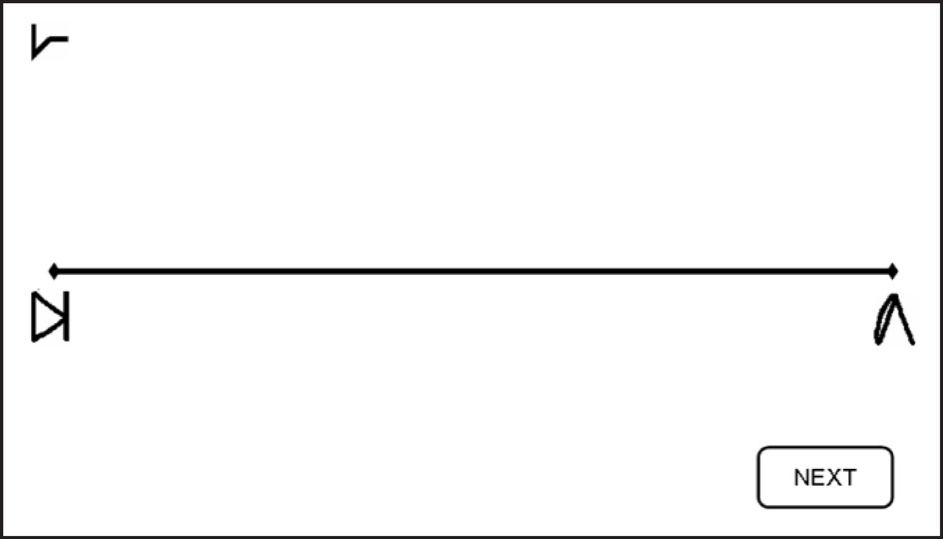
**Number Line task**. Subjects were asked to map the given symbol, which appeared randomly at the left upper corner—as in the current example—or at the right upper corner, on the physical line. Subjects were instructed to place each symbol on the line according to its magnitude. Reprinted from Cohen Kadosh et al. (2010), with permission from Elsevier.

In addition to the learning task, sessions two through six (Figure 2) included a numerical Stroop task and a number line task (Figure 3).

***Numerical Stroop tasks*.** In the numerical Stroop tasks (Figure 2C) the artificial digits appeared on the screen in the same manner as in the learning task, but the symbols were different in physical size (vertical visual angle of 2.2° or 2.75°). Subjects were instructed to choose the *physically* larger (Numerical Stroop task) symbol by pressing either P or Q buttons as quickly and accurately as possible. While all the possible adjacent pairs were used (e.g., 1–2, 2–3, 3–4) in the learning phase (Figures 2A,B) only non-adjacent pairs were used here (e.g., 1–3, 2–4, etc.) (Figure 2C) and were divided to small numerical distance (numerical distance of 2 units, e.g., 2–4, 5–7) or large numerical distance (numerical distance of 5 units, e.g., 2–7, 3–8), and congruent, incongruent, and neutral conditions were included in order to examine the possible generation of automatic numerical representations (Tzelgov et al., 2000). In a congruent pair, the numerically larger artificial digit was also physically larger. In a neutral pair, the digits differed only in the relevant dimension (i.e., size). In an incongruent pair, the numerically larger digit was physically smaller. The artificial digits that were the equivalent to the numbers 1 and 9 received the same classification during the learning phase (small, and large, respectively) and were not included in the analysis (Tzelgov et al., 2000). The three conditions appeared the same number of times, with the right answer appearing equal times on the right and left sides and all pairs appearing equally often. No feedback was given.

***Number line task*.** In the number line task, participants had to map symbols (i.e., the artificial digits) onto a horizontal line displayed on the computer screen. The symbol corresponding to number “1” was placed at the left-end of the line, and the symbol representing number “9” at the right-end of the line (Figure 3). Subjects were instructed to place each of the remaining seven symbols on the line according to their magnitude. Symbols to be mapped appeared above the right- and left-end of the line in a randomized order to avoid any bias in responses that might arise due to stimulus location (Nichelli et al., 1989). Each symbol appeared 3 times at each location, making 42 line bisection trials in total for each session. No feedback was given.

#### Procedure

The study consisted of six sessions for each subject. The sessions lasted ~120 min each (including electrode placement, the learning phase, and the testing phase) and were distributed over a 7-day period. Each subject attended one session per day apart from a break after the 4th day (Cohen Kadosh et al., 2010) (Figure 2). The first session only consisted of the learning task for both subjects. During the remaining sessions (2–6) the learning task plus the two experimental tasks (i.e., Stroop-task and number line task) were administered to each subject (Figure 2).

### tDCS protocol

We chose to use tDCS, the most frequently used application of tES. This technique delivers low electric current to the scalp to modulate the resting membrane potentials of underlying neurons by hyperpolarizing them (cathodal stimulation) or partially depolarizing them (anodal stimulation). Direct current was generated by a Neuroconn stimulator (Ilmenau, Germany) and delivered via a pair of identical, rectangular, scalp electrodes (3 × 3 cm) covered with conductive rubber and saline soaked synthetic sponges.

For both participants, at the beginning of the stimulation the current was increased slowly during the first 15 s to the stimulation threshold (1 mA) (ramp-up), and at the end of the stimulation the current was decreased slowly to 0 mA during the last 15 s (ramp-down). Between the ramp-up and ramp-down constant direct current (1 mA) was delivered for 20 min at the beginning of each session. Electrodes were positioned over the left and right posterior parietal lobes according to the 10–20 EEG procedure on the sites corresponding to P3 and P4 respectively. We chose to place the cathodal electrode on the controlateral side of the parietal lobe, and not on the prefrontal cortex, not to affect the mechanisms that might relate to learning (Iuculano and Cohen Kadosh, 2013), and feedback/reward, which was provided during the learning phase (Duncan, 2001; Albert et al., 2009). Other brain areas that might be involved in visual, semantic or numerical processing (occipital lobes, temporal lobes) were also excluded (Cohen Kadosh and Walsh, 2009). In addition, the placement of the electrodes over both parietal lobes increases the specificity of the type of stimulation to each lobe, and maximized its effect by increasing the current density (Nathan et al., 1993).

Although stimulation ended during the learning task, electrodes were kept in place until the task was completed in order to avoid participant's bias. Participants reported a slight tingling sensation during the stimulation, which diminished rapidly due to habituation. No other discomforts or adverse effects were reported.

#### tDCS conditions

In line with our previous study (Cohen Kadosh et al., 2010) participants were randomly assigned to one of two stimulation conditions: (i) Right Anodal-Left Cathodal (RA-LC)—DD1 received excitatory (anodal) stimulation to the right PPC, and inhibitory (cathodal) stimulation to the left PPC for 20 min per day; (ii) Left Anodal-Right Cathodal (LA-RC)—DD2 received excitatory (anodal) stimulation to the left PPC, and inhibitory (cathodal) stimulation to the right PPC for 20 min per day. Subjects were informed that the experiment was designed to investigate effects of tDCS on cognition but were kept blind as to the specific relevance to numerical cognition and to the type of stimulation they were receiving.

## Results

### Learning task

The learning of each participant was assessed by fitting their performance using the following power law function (Newell and Rosenbloom, 1981):

$$RT=B*{\left(N\right)}^{-C}$$

**Figure 4:**
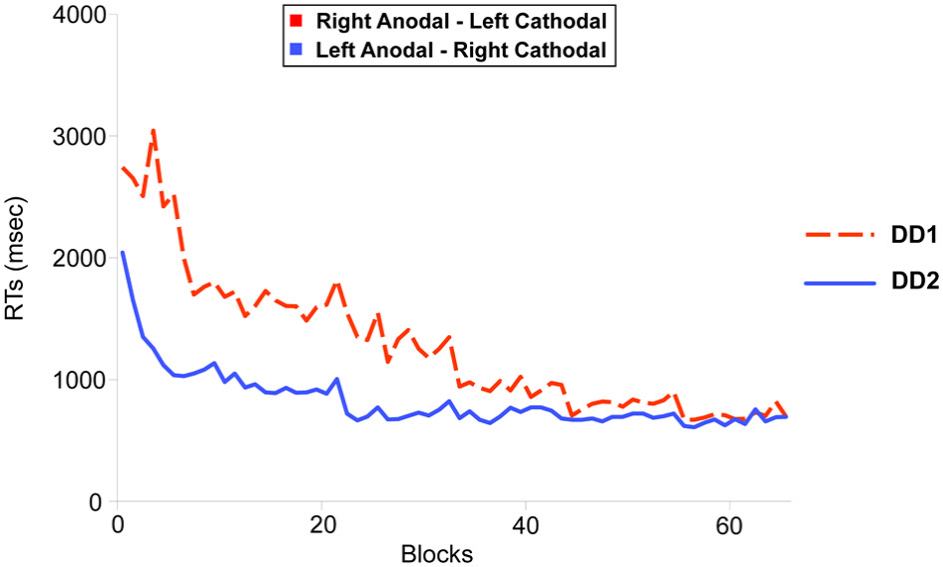
**Learning functions for the two DD individuals**. DD1 received Right Anodal—Left Cathodal (RA-LC) stimulation to the PPC (dotted red line); DD2 received Left Anodal—Right Cathodal (RC-LA) stimulation to the PPC (solid blue line). The improvement in the learning task over blocks (x-axis) was modeled using a power law function. Non-linear regression showed an equivalent fit for both participants (RA-LC, *R* = 0.9; RC-LA, *R* = 0.96).

In this equation, *RT* represents the mean Reaction Time in a given block, *B* is the performance in time on the first block (*N* = 1), N the number of the block and *C* represents the slope of the line (i.e., the learning rate). Non-linear regressions showed a similar fit for both participants (LA-RC, *R* = 0.96; RA-LC, *R* = 0.9 (Figure 4); which is comparable to the fit shown by neuro-typical adult participants in Cohen Kadosh et al. (2010): LA-RC, *R* = 0.92; RA-LC, *R* = 0.88).

### Experimental tasks

#### Numerical stroop tasks

**Figure 5:**
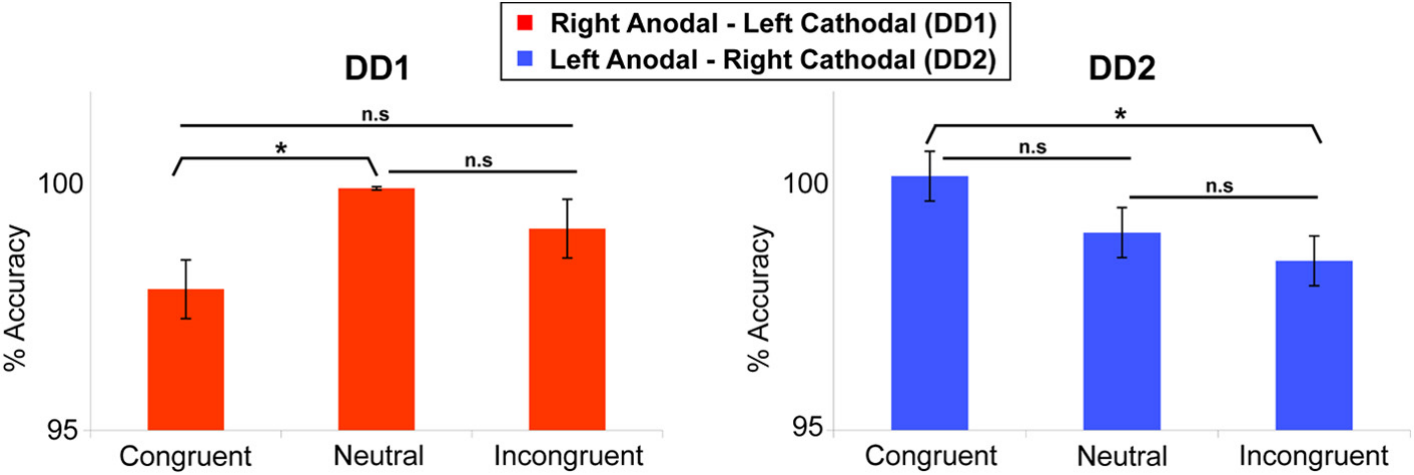
**Numerical Stroop task**. Congruency effect (measured in terms of accuracy) for the two DD individuals. DD1 did not exhibit the canonical Congruency effect (Congruent > Neutral > Incongruent), while DD2 showed a clear Congruency pattern in the predicted direction. For DD1: Neutral > Congruent (*p* < 0.05); Congruent vs. Incongruent (*p* = 0.13); Incongruent vs. Neutral (*p* = 0.08). For DD2: Congruent > Incongruent (*p* < 0.05); Congruent vs. Neutral (*p* = 0.07); Incongruent vs. Neutral (*p* = 0.16). Data are mean ± standard error (SE) of the mean. ^*^p < 0.05.

DD1's performance was better on Neutral trials as compared to Congruent trials (*t*_(239)_ = 2.255, *p* < 0.05). No differences were found on the other comparisons (*p* = 0.13 and *p* = 0.08 for Congruent versus Incongruent; and Incongruent versus Neutral trials respectively) (Figure 5).

In contrast, for DD2, the results showed the clear emergence of a canonical congruency effect in the predicted direction: Congruent trials were more accurate than Incongruent trials (*t*_(239)_ = 1.739, *p* < 0.05) (Figure 5), while the other comparisons did not show any significant effect (*p* = 0.07 and *p* = 0.16 for Congruent versus Neutral; and Incongruent versus Neutral trials respectively).

To look at the results in more details, performance on the numerical Stroop task was analyzed in terms of reaction times (RTs) using a two-way analysis of variance with Numerical Distance (Small, Large) and Congruency (Congruent, Neutral, Incongruent), independently for each of our DD cases. RTs below 150 ms and above 1500 ms were excluded from the analyses (3.6% of the data).

**Figure 6:**
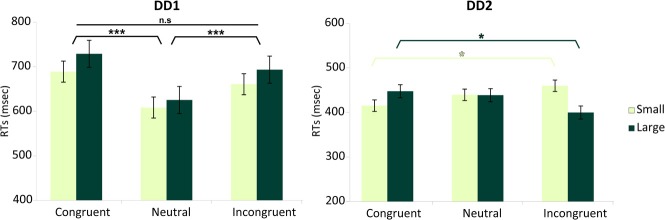
**Numerical distance by congruency effects**. Effects measured in terms of RTs for each DD individual. DD1 did not exhibit the canonical Congruency effect (Incongruent > Neutral > Congruent), while DD2 showed a Congruency pattern related to the numerical distance between stimuli. For DD1 both Congruent as well as Incongruent trials were slower than Neutral trials (*p* < 0.001) and no effect of numerical distance was evident [*F* < 1]. In DD2, the canonical pattern typical of the Congruency effect (Incongruent > Congruent) was only present for small distances (e.g., 2–4) (*p* < 0.05); while the reverse pattern (Congruent > Incongruent) characterized DD2's performance with large numerical distances (e.g., 2–7) (*p* < 0.05). Main effects are shown in black (DD1's profile). Interaction is shown in shades of green (DD2's profile). Data are mean ± standard error (SE) of the mean. ^*^p < 0.05; ^***^p < 0.001.

***DD1—RA-LC*.** For DD1, the analysis revealed no main effect of Numerical Distance [*F* < 1], while there was a main effect of Congruency [*F*_(2, 358)_ = 9.61, *p* < 0.0001]. Specifically, Incongruent and Congruent trials did not differ from each other [*F*_(1, 358)_ = 1.27, *p* > 0.26], but they both differed from the Neutral condition [*F*_(1, 358)_ = 18, *p* < 0.001] (Figure 6). The interaction Numerical Distance by Congruency was not significant [*F*_(2, 358)_ = 0.74, *p* > 0.93].

***DD2—LA-RC*.** For DD2, none of the main effects was significant: Numerical Distance [*F* < 1], Congruency [*F* < 1]. However, there was a significant interaction Numerical Distance by Congruency [*F*_(2, 383)_ = 5.65, *p* < 0.004]. Decomposing congruency according to Small and Large distances revealed significantly slower reaction times for Incongruent compared to Congruent trials for Small distances [*F*_(1, 383)_ = 5.14, *p* < 0.023]; while the reverse pattern was observed for Large distances (i.e., slower reaction time for Congruent compared to Incongruent trials) [*F*_(1, 383)_ = 5.8, *p* < 0.016] (Figure 6).

#### Number line task

**Figure 7:**
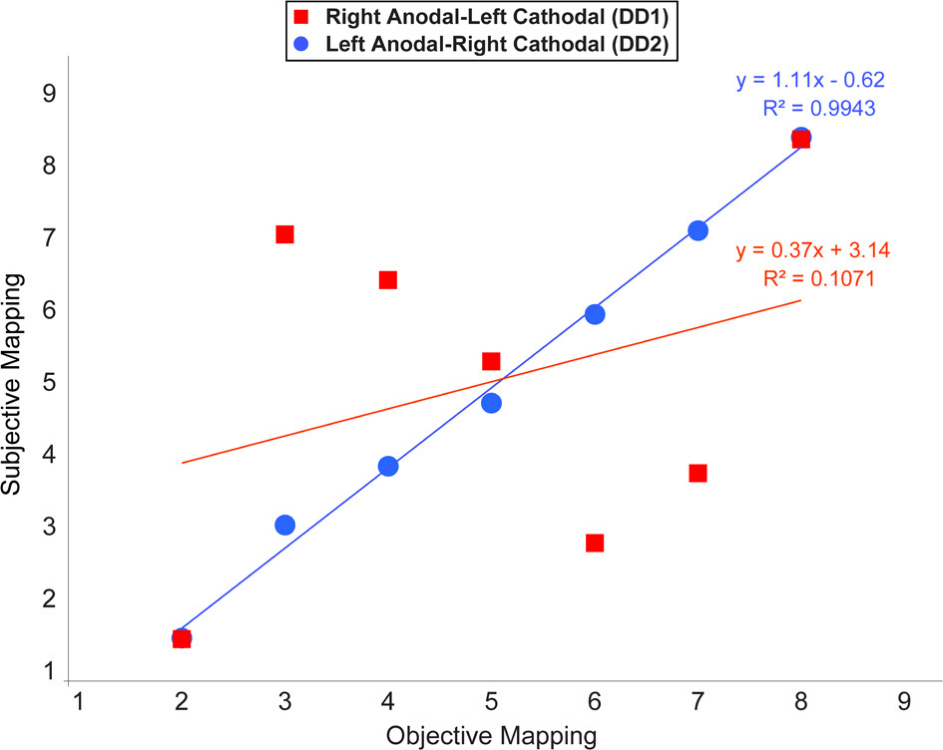
**Average location of subjective responses on the number line task plotted for each type of stimulation**. Linear regression lines and equations are indicated for each type of stimulation (Red line—Right Anodal-Left Cathodal stimulation received by DD1; Blue line—Left Anodal-Right Cathodal stimulation received by DD2).

In this analysis we examined whether the mapping of numbers onto space followed a linear scale. Notably, at the end of the learning, a linear function was the best predictor in the case of DD2. In contrast, the pattern of results characterizing DD1 was rather random, except for the symbols representing the numbers 2 and 8 (Figure 7).

## Discussion

In this study, we assessed whether brain stimulation to the PPC coupled with a learning paradigm could affect numerical competence in two adult individuals with DD. In order to simulate the cognitive process that characterizes the learning and subsequent mastering of new numerical information as it occurs during the early stages of development, we created a new numerical system using artificial symbols—i.e., the Gibson figures (Gibson et al., 1962; Tzelgov et al., 2000; Cohen Kadosh et al., 2010)—participants had to learn the implicit association between the artificial symbols and their corresponding—and arbitrarily assigned—numerical magnitude, while brain stimulation was delivered to the PPC using tDCS. The polarity (i.e., anodal or cathodal) of the stimulation was a function of brain laterality (i.e., left or right PPC) and differed between the two DD participants. We found that the polarity of the brain stimulation could enhance the acquisition of automatic number processing—as assessed by the numerical Stroop task —, as well as the mapping of numbers onto space—as assessed by the number line task —, both considered reliable indices of numerical proficiency (Girelli et al., 2000; Rubinsten et al., 2002; Booth and Siegler, 2008; Dehaene et al., 2008). Specifically, anodal stimulation and concurrent cathodal stimulation to the left and right PPC respectively, boosted numerical performance in one of our participants (DD2); while in the other participant (DD1), the opposite configuration (i.e., Right Anodal—Left Cathodal) did not lead to any performance improvement.

Developmental Dyscalculia is a very debilitating learning disability that affects between 3 and 7% of the population, and has a serious impact on the educational, professional but also psychological outcomes of the individuals affected (Parsons and Bynner, 2005; Butterworth et al., 2011). Research focused on the intervention approaches to help DD individuals to overcome their difficulties has been promising, but it is still in its infancy. Thus far, this type of research has focused on behavioral remediations and has almost solely targeted developmental populations (Dowker, 2009). Little is currently known on whether remediation can occur later in development, namely in adult individuals with DD. Even more importantly, to the best of our knowledge nothing is currently known about the effectiveness of the concurrent application of brain-behavior rehabilitation approaches. To our knowledge, this is the first study to examine the potential remediational effects of tES-*like* techniques in adults with DD.

Our findings indicate that one of the two possible configurations of bilateral stimulation to the PPC (i.e., Left Anodal—Right Cathodal), can lead to significant performance improvements on both indices of numerical proficiency. Following the learning of the association between pairs of symbols that appeared only adjacently DD2—who received Left Anodal—Right Cathodal tDCS—was able to make the required transitive inference from adjacent pairs to non-adjacent pairs, and understand the ordinality as well as cardinality properties of the new symbolic system. Namely, as reflected by her performance on the number line task, DD2 generated an accurate representation of the artificial digits; while DD1—who received Right Anodal—Left Cathodal tDCS—did not (Figure 7). Signs of a successful performance after Left Anodal—Right Cathodal, but not Right Anodal—Left Cathodal tDCS, is further supported by the results on the numerical Stroop task. When presented with non-adjacent pairs, DD1 did not show any evidence of transitive inference, as she did not exhibit a congruency effect (Figure 5). In contrast, DD2 showed a congruency effect on the accuracy variable (Figure 5). Moreover, DD2 showed a significant congruency effect also in terms of reaction times (Figure 6). Yet, in this case, the effect was in the opposite direction to the one observed in neuro-typical adults (Schwarz and Ischebeck, 2003; Cohen Kadosh et al., 2007b, 2008). Namely, the effect characterizing the performance of DD2 was stronger and in the expected direction for small numerical distances, and reversed for large numerical distances. This pattern of results might reflect different strategy use by DD2, which might have developed to compensate for a deep rooted magnitude-based deficit (Butterworth, 2005). A likely compensatory strategy could include verbally-mediated approaches occurring during the learning phase. Contrarily, neuro-typical adults might rely more on a purely magnitude-based system—anchored in the right parietal lobe—when solving this task (Cohen Kadosh et al., 2007a, 2010). Indeed, the latter possibility might explain the discrepancy between site of stimulation and performance in typical and atypical participants. Namely, using the same learning paradigm, as well as tDCS montage with three stimulation groups (Left-Anodal, Right-Cathodal, Right-Anodal, Left-Cathodal, and Sham) in typically developing adults, we found that Left-Anodal, Right-Cathodal stimulation (i.e., the same montage applied to DD2) was instead characterized by a significant impairment or no improvement in the number line task, and no automaticity effect, similar to the pattern exhibited by DD1. In contrast, when the opposite configuration was applied to typically developing adults—Right Anodal-Left Cathodal—(i.e., the montage applied to DD1) performance enhancement occurred—as indicated by a linear mapping of the artificial digits in the number line task, as well as automatic processing of the learned symbols. These apparent discrepancies in the directionality of the results might have different explanations related to stimulation site and its interactions with the contralateral site of stimulation: (i) it is possible that the effect of the brain stimulation depend on the initial neural activation-i.e. state- of the subject (see Krause et al., 2013), so that if the stimulated area is normally involved during the cognitive task, anodal stimulation might lead to cognitive enhancement of that function, while cathodal stimulation will not be effective as the neurons are already activated in the given task. This hypothesis might explain the pattern found in neuro-typical adults (Cohen Kadosh et al., 2010), as well as the null-result reported for DD1, for whom the targeted site (i.e., the right IPS) might not be already active during the task; (ii) another possibility is that cathodal stimulation to the impaired brain region (i.e., right IPS in this case), will not lead to performance impairment, as the contralateral brain region will show tDCS-contingent compensation for the reduction in the stimulated brain region's excitability (see for example Jacobson et al., 2012); (iii) it is finally plausible that compensatory neural mechanisms as well as strategy variability in our DD subjects might have taken place. Notably, as it seems to be the case for neurological patients, it is conceivable that in cases where DD persists into adulthood, neural, as well as cognitive compensatory mechanisms are taking place. Indeed, the behavioral outcome in DD2 is different from the one expected in neuro-typical adults (Figures 5, 6), suggesting that differential strategies might be adopted by the subject while solving the task. Furthermore, is possible that the system to be boosted through tES shifts hemispheres. That is, from a right PPC-based system that is specialized in magnitude representation, to a left PPC-system that relies more on a verbal and mnemonic code (Dehaene et al., 2003). Indeed, a signature for neural re-organization has been proposed for DD children as young as 9 years old (Kaufmann et al., 2009). Interestingly, these authors specifically attribute it to a mechanism of inter-hemispheric compensation from right to left PPC. Notably, it has been empirically demonstrated that neural circuits have the potential for re-modeling themselves in the contralateral hemisphere during functional recovery from cerebral infarction (Takatsuru et al., 2009). Furthermore, and because of such neural re-organization, it is important to note that neuroplastic effects elicited by tDCS might obey different neural constraints in atypical populations, as compared to neuro-typical individuals. Thus, for the patient group, tDCS will affect cortical function by modulating inter-hemispherical interactions, whereby upregulating the excitability of the compensatory neural populations (i.e., in the left PPC), while downregulating that of the right PPC, would decrease inter-hemispheric inhibition (Ferbert et al., 1992) and thereby improve performance. Notably, such mechanistic phenomenon has been reported to occur during stimulation of the motor cortices (Vines et al., 2006), but also of the PPC during visual processing (Battelli et al., 2009), as well as numerical tasks (Hauser et al., 2013). Critically, tES studies on neurological patients have demonstrated that this technique might reveal its best results when it is acting on the proposely imbalanced inter-hemispheric interactions (Lindenberg et al., 2010) that likely occur after a stroke (Murase et al., 2004, for a recent review see Zimerman and Hummel, in press). Together, these studies seem to highlight the fundamental differences of tES application in respect of the type of population under study (i.e., neuro-typical vs. clinical subjects).

In conclusion, this study adds to the emerging literature looking at possible intervention approaches to help DD individuals with their difficulties, and points to a new potential treatment tool as a viable option for brain-behavior rehabilitation. While this study provides just a proof of concept, several limitations need to be discussed. First, further studies with larger sample sizes are needed, to better control for the issue of individual differences in DD. Namely, inasmuch as our DD cases were carefully selected and were indeed critically impaired in various numerical and arithmetical tasks, performance on different tasks, while being similar could have not been fully matched (see Table 1). Thus our current data leaves open the question of whether it would have been possible to enhance performance of DD1 as well, if the successful tES configuration (i.e., Left Anodal-Right Cathodal) was applied instead. Alternatively, it is possible that the different results obtained for our two DDs might have depended on the patient's individual morphology, or the severity or type of the deficit, further highlighting the fact that especially with clinical populations, the optimal stimulation approach—including site of stimulation, electrodes montage, duration of the stimulation, current intensity, etc.—needs to be decided on a patient by patient basis (Truong et al., in press). Furthermore, it is important to note that our study did not test longevity nor transfer effects, which are crucial aspects of rehabilitation protocols, and could therefore better establish this technique as a promising rehabilitation tool for this condition. Further studies are also needed which implement simultaneous tES-fMRI/EEG paradigms, to properly examine the hypothesis of neural inter-hemispheric re-organization for cases of persistent developmental learning disabilities such as DD. The combination of such techniques will also be able to inform on the neural mechanisms through which tES operates by, and specifically whether they might actually reflect the ones proposed here. Last, the use of more advanced types of tES, such as transcranial random noise stimulation (tRNS), which has been shown to enhance arithmetic learning and performance, can be useful (Snowball et al., 2013). Compared to tDCS, tRNS is polarity-independent, and can therefore excite both hemispheres. Thus, it might offer a more attractive method for stimulation, especially in cases where there is relative lack of knowledge on cortical re-organization (Cohen Kadosh et al., 2013).

Our findings represent an important initial step toward a new line of research that could contribute to establish effective treatments which may potentiate cerebral adaptive processes and thereby facilitate the rehabilitation of DD cases.

### Conflict of interest statement

Roi Cohen Kadosh filed a patent for an apparatus for improving and/or maintaining numerical ability. The other author declares that the research was conducted in the absence of any commercial or financial relationships that could be construed as a potential conflict of interest.
